# Native and Non-Native Speakers’ Recognition of Chinese Two-Character Words in Audio Sentence Comprehension

**DOI:** 10.3390/bs14121169

**Published:** 2024-12-06

**Authors:** Wenling Ma, Degao Li, Xiuling Dong

**Affiliations:** College of Chinese Language and Literature, Qufu Normal University, No. 57, Jingxuan Road, Qufu 273165, China; wenling_ma@qfnu.edu.cn (W.M.); dongxiuling@qfnu.edu.cn (X.D.)

**Keywords:** native speakers, non-native speakers, recognition, Chinese two-character words, sentence comprehension

## Abstract

Two experiments were conducted to examine native and non-native speakers’ recognition of Chinese two-character words (2C-words) in the context of audio sentence comprehension. The recording was played of a sentence, in which a collocation composed of a number word, a sortal classifier, and a noun (NCN) was embedded. When the participants were about to hear the noun of the NCN (Noun), the playing stopped, and a target was visually presented, which was the Noun, the character-transposed word of the Noun (NounT), or a control word (NounC), or was a homophone nonword for Noun, NounT, or NounC. The participants were required to make a lexical decision on the target before they resumed listening. The results showed that both native and non-native speakers were able to take visually presented 2C-word targets as semantic whole entities in the context of audio sentence comprehension, which was mediated by their Chinese proficiency. Native speakers readily processed visually presented 2C-words both as wholes and according to their constituent characters, but non-native speakers were not likely to process the 2C-words according to their constituent characters.

## 1. Introduction

In Chinese, the graphic units are Chinese characters. Most of the commonly used characters are often joined with others to form two-character words (2C-words) or words of more than two characters. By words, we mean the items that are listed in an ordinary Chinese word dictionary. Indeed, over 70% of the most frequently used words [1] are 2C-words. An orthographic feature of 2C-words is that the constituent characters have fixed relative positions. If the positions of the constituent characters of a 2C-word are exchanged, there is a 96.7% chance of creating a character-transposed nonword, i.e., a cluster of two characters that does not form a recognized word [2]. However, in the early stage of 2C-word recognition, the processing of the positional information of constituent characters may not be necessarily inevitable [3,4,5].

In a priming lexical decision task, for example, the processing of a 2C-word (e.g., ‘领带’, /ling3dai4/, *tie*) or its character-transposed word (e.g., ‘带领’, /dai4ling3/, *to lead*) had the same size of priming effect on the participants’ responses to the target (e.g., ‘西装’, /xi1zhuang1/, *suit*) at a stimulus onset asynchrony (SOA) of 157 ms [3]. There are about 800 commonly used 2C-words that turn into other words upon character transposition [6]. Briefly, simultaneous presentations of the constituent characters (e.g., ‘带’, /dai4/, *belt*; ‘领’, /ling3/, *collar*), whether in the form of a 2C-word or its character-transposed word, helped to activate the representations for the target, which was semantically associated with the word prime but not with the character-transposed-word prime. Du et al. [4] adopted a delayed repetition priming paradigm using the event-related potential (ERP) technique and asked participants to passively view a list of 2C stimuli that contained prime–target pairs. The prime and target were identical 2C-words (e.g., ‘经理’, /jing1li3/-’经理’, /jing1li3/, *manager*) or were a character-transposed nonword and its corresponding word (e.g., ‘士护’, /shi4hu4/-’护士’, /hu4shi0/, *nurse*). An N400 was observed that did not differ in amplitude between the two types of prime–target pairs. N400 indicates a violation in semantic processing [7]. In a priming semantic categorization task, participants’ responses to the targets (e.g., ‘香蕉’, /xiang1jiao1/, *banana*) were similarly facilitated when the primes were 2C-words (e.g., ‘苹果’, /ping2guo3/, *apple*) and the character-transposed nonwords (e.g., ‘果苹’, /guo3ping2/) [5].

There is also evidence that a 2C-word can be perceived as an orthographic whole. In Peng, Ding et al. [3] for example, participants’ responses to targets (e.g., ‘西装’, /xi1zhuang1/, *suit*) were facilitated when the primes were words (e.g., ‘领带’, /ling3dai4/, *tie*) rather than transposed words (e.g., ‘带领’, /dai4ling3/, *to lead*), at SOAs of 57 ms and 314 ms. Several theoretical models have been established concerning 2C-word representations [8,9,10], which all predict that 2C-words in isolation are likely to be processed both as whole entities and according to their constituent characters.

Since words are the basic units in sentence reading [11], one may wonder whether a 2C-word can be processed according to its constituent characters in the context of sentence comprehension. To the authors’ knowledge, however, not much evidence seems to be available on this point. In addition, each Chinese character corresponds to a syllable in spoken Chinese. Gradually and with regular practice, learners may establish a strong association between a word and its pronunciation as a whole. For skilled readers, the phonological processing of a 2C-word might occur through the rapid activation of the whole representation [12]. Thus, there may be some interaction between orthographic and phonological processing in 2C-word recognition in the context of sentence comprehension, which should be mediated by the readers’ Chinese proficiency. The present study attempts to test this proposal using a lexical decision task in audio sentence comprehension (LDA) [13].

### 1.1. Literature Review

Swinney et al. [13] played the audio recording of a sentence in which an adjective–noun collocation (e.g., ‘peeled banana’) was embedded. When the participants were about to hear the noun (e.g., ‘banana’), the audio stopped and a word target (i.e., ‘yellow’ or ‘white’), which suggested a property of the noun of the collocation, was visually presented on the computer screen. The target was presented for 300 ms and then the participants were required to decide whether or not it was a meaningful word. Having significantly shorter reaction times (RTs) for ‘yellow’ than for ‘white’, the participants must have activated the representation for the noun ‘banana’ when hearing the adjective ‘peeled’. This means that processing the beginning part of a collocation helps to activate the representation for the following part in the context of audio sentence comprehension.

There is a particular collocation in Chinese composed of a number word, a sortal classifier (classifier), and a noun; hereafter referred to as an NCN. For example, ‘一张桌子’ (/yi1zhang1 zhuo1zi0/, *one table*) consists of the number word ‘一’ (/yi1/, *one*), the classifier ‘张’ (/zhang1/), and the noun ‘桌子’ (/zhuo1zi0/, *table*). Similarly, the words in ‘三本书’ (/san1ben3shu1/, *three books*) are the number word ‘三’ (/san1, *three*), the classifier ‘本’(/ben3/), and the noun ‘书’ (/shu1/, *books*). The noun is collocated with the classifier, which carries specific information. For example, ‘一本书’ (/yi1ben3shu1/, *a book*) is different from ‘一摞书’ (/yi1luo4shu1/, *a stack of books*). The classifier in the former indicates a kind of thing that looks like a book, but the classifier in the latter suggests the configuration of three or more book-like things with one above another. To a certain extent, the noun determines the classifier in an NCN. For example, the blank in ‘一 树’(/yi1 shu4/) can only be filled with the classifier ‘棵’ (/ke1/) or ‘排’ (/pai2/). ‘棵’ (/ke1/) means a single plant and ‘排’ (/pai2/) means a line of things that stand like trees.

The classifier provides a strong cue for the expectation of the upcoming noun in an NCN in sentence comprehension. For example, an NCN (e.g., ‘一贴膏药’, /yi1tie1 gao1yao4/, *a piece of plaster*) is embedded in a sentence (e.g., ‘妈妈有点腰疼, 叫我帮她在腰上贴了一贴膏药, 但是它的效果不是很好’, /ma1ma0 you3dian3 yao1teng2, jiao4 wo3 bang1ta1 zai4 yao1shang0 tie1le0 yi1tie1 gao1yao4, dan4shi4 ta1de0 xiao4guo3 bing4 bu2shi4 hen3hao3/, *Mum had a backache and asked me to put a piece of plaster on her waist, but it didn’t work very well*). After the participants hear the audio recording of the pre-text (e.g., ‘妈妈有点腰疼, 叫我帮她在腰上贴了一贴’, /ma1ma0 you3dian3 yao1teng2, jiao4 wo3 bang1ta1 zai4 yao1shang0 tie1le0 yi1tie1/, *Mum had a little backache and asked me to put a piece of*), the playing stops and the target stimulus is visually displayed on the computer screen. This stimulus is either the noun of the NCN (e.g., ‘膏药’, /gao1yao4/, *plaster*) (Noun), the character-transposed word of the Noun (e.g., ‘药膏’, /yao4gao1/, *ointment*) (NounT), or a control word (e.g., ‘公鸡’, /gong1ji1/, *cock*) (NounC). Participants are required to decide whether or not the target is a meaningfully acceptable word. The key test is whether they perform similarly in the cases of Noun and NounT (Research Question One).

The pronunciation of a 2C-word (e.g., ‘捷径’, /jie2jing4/, *shortcut*) is often the same as that of another word (e.g., ‘洁净’, /jie2jing4/, *hygienic*) in Pinyin, the character pronunciation system in modern Chinese. Taking advantage of the similarity between words in pronunciation, researchers have conducted studies on phonological processing in 2C-word recognition. In a word-by-word self-paced reading task [14], Wang and Li [12] found that participants tended to confuse a 2C-word with its homophone in sentence reading. For example, in ‘他经常在傍晚独自欣赏夕阳照亮天空’ (/ta1 jing1chang2 zai4 bang4wan3 du2zi4 xin1shang3 xi1yang2 zhao4liang4 tian1kong1/, *he often enjoys the setting sun by himself at dusk*), the target word ‘夕阳’ (/xi1yang2/, *the setting sun*) in the second part of the sentence is semantically related to the prime word ‘傍晚’ (/bang4wan3/, *at dusk*) in the first part. Participants had significantly shorter reading times when the target word was replaced with its homophone (e.g., ‘西洋’, /xi1yang2/, *occident*) than when it was replaced with a control word (e.g., ‘廉价’, /lian2jia4/, *cheapness*).

Newman and Connolly [15] required participants to read a highly constrained sentence, the last word of which took one of four forms: (a) phonologically, orthographically, and semantically appropriate (e.g., ‘The gambler had a streak of bad luck’); (b) a homophone nonword orthographically incongruent but phonologically congruent to the anticipated ending (e.g., ‘New York is a very busy sitee’); (c) orthographically, phonologically, and semantically incongruent to expectations (e.g., ‘The dog chased the cat up the Queen’); (d) a nonword that was orthographically and phonologically incongruent to expectations (e.g., ‘The gas station is about two miles down the bole’). The ERP measurement of the participants’ processing of the last word indicated that the amplitude of N400 was similarly reduced in the first two conditions compared with the third or fourth conditions. On the encounter of a meaningless string (e.g., ‘sitee’), participants could not access a meaning using the orthographic units. This shows that the activation of the corresponding phonological units is essential as they produce feedback activation of the correct orthographic units (e.g., ‘city’), allowing access to the context-appropriate meaning.

In short, both Wang and Li [12] and Newman and Connolly [15] found that the sentential context seemed to help trigger the phonological processing of the target words. The mechanism of language comprehension in reading is similar to that in listening [16]. In the present study, if homophone nonwords are adopted as no-response targets in the Noun, NounT, and NounC conditions in the LDA, the key test is whether participants find it more difficult to make ‘no’ responses in the Noun condition than in the NounT or NounC conditions (Research Question Two).

Adopting a repetition priming paradigm, Gao et al. [17] asked participants to make lexical decisions on 2C-word targets (e.g., ‘天气’, /tian1qi4/, *weather*) that were preceded either by the targets themselves (e.g., ‘天气’, /tian1qi4/, *weather*) or by one constituent character of the targets (e.g., ‘天’, /tian1/, *sky*). Both native speakers and advanced-level non-native speakers of Chinese showed a significantly better performance in the whole word primer condition than in the constituent character primer condition. Therefore, the whole word might be the optimal unit for storage and retrieval of 2C-words for these two groups of participants. However, a similar size of the priming effect was observed for intermediate-level non-native speakers, who might have processed 2C-words either as whole entities or according to their constituent characters.

Considering this study together with those by Peng, Ding et al. [3], Du et al. [4], and Wang et al. [5], it may be concluded that readers’ 2C-word recognition is subject to the mediation of their proficiency and language experiences. Particularly, Gao et al. [17] argued that second-language (L2) Chinese learners of an intermediate level could process 2C-words both as whole entities and according to their constituent characters. Given their lack of experience compared with native speakers, however, the intermediate L2 Chinese learners should have developed a relatively limited vocabulary in the target language. Their perception of the one-character prime (e.g., ‘天’, /tian1/, *sky*) might be more likely than that of the native speakers to initiate representation activation only for the target (e.g., ‘天气’, /tian1qi4/, *weather*). However, the native speakers’ processing of the prime (e.g., ‘天’, /tian1/, *sky*) might have initiated the activation of representations for many 2C-words, which contain the prime as the constituent character on the left. According to Cai and Brysbaet [18], for example, there are 112 2C-words with ‘天’(/tian1/, *sky*) as the initial constituent character. If this argument is plausible, then the key question to explore is how language proficiency affects participants’ performance in the LDA (Research Question Three).

### 1.2. Present Study

Adult native speakers of Chinese may fall into more than one category in terms of their language competence. For example, in the college entrance examination, students in a university in Xinjiang, China (HanLs) do not seem able to achieve as high scores in Chinese as those in a university in Shandong, China (HanHs). This is probably due to differences in their motivation for learning and in peer competition in pre-college education.

In addition to the Han majority, there are 55 ethnic minorities recognized in China. Young people from these minority groups (except those from the Hui, Manchu, and She people who use Chinese as their first language) learn Chinese as L2. One example is the 10 million Uyghurs in China, the majority of whom inhabit Xinjiang. In elementary schools, Uyghur students’ practice in Chinese seems to be strictly limited to classroom activities. In primary and secondary education, both Chinese and Uyghur are adopted as language media. In colleges, Chinese is the main language in classroom activities, but outside classrooms, Uyghur students communicate with each other in their mother tongue and prefer to read books and access media in Uyghur. This inadequate immersion in Chinese means that Uyghur students are slower to master the target language. Indeed, Uyghur students in the first year of primary school have a smaller Chinese vocabulary than Han students in grade two of elementary school [19]. After at least 12 years of school education, however, Uyghur college students are able to undertake courses in Chinese.

Another example is the Korean population, who are the predominant ethnic minority in north-eastern China. In contrast with Uyghurs, who are from communities that are physically separate from the Han Chinese communities, more than half of the Korean minority group live in the same communities as Han Chinese. Korean students are able to use both Korean and Chinese inside and outside the classroom and are able to read books and access media in both languages. They tend to have the same opportunity to use Chinese as they use Korean in everyday activities. Moreover, more than 60% of Korean words are borrowed from Chinese. The written form of a Korean syllable assumes a square-shaped space similar to the written form of a Chinese character. For example, the written word for school is ‘학교’ in Korean. The first (‘학’) and second (‘교’) syllables correspond to the first (‘学’, /xue2/, *to learn*) and second (‘校’, /xiao4/, *school*) constituent characters of the 2C-word for school. This orthographic similarity may make Korean learners see a 2C-word as being composed of two Chinese characters.

The present study therefore included two experiments, each of which adopted a design of a 2 (sbjGroup) × 3 (trgCondition: Noun, NounT, or NounC) × 2 (trgType: Word or Nonword) factorial of mixed measurements, with trgCondition and trgType as the repeated variables. The two levels of sbjGroup were HanHs and HanLs (Experiment 1) and Koreans and Uyghurs (Experiment 2). The dependent variables were participants’ response errors and correct response RTs in the LDA. By comparing the influence of Chinese proficiency on native and non-native speakers’ performance, the present study attempted to address the three research questions outlined above.

If the proficiency differences of Chinese non-native speakers interact with trgCondition and trgType similarly to how those of Chinese native speakers do, then the two experiments will yield similar patterns of results according to the following predictions:

(1) Participants are able to predict the Noun prior to its appearance, and a ‘yes’ response is easier to make when the target is a Noun than when it is a NounC;
(2) Participants are able to process the target according to its constituent characters, and they will achieve a similar pattern of performance between the Noun and NounT targets but not between the Noun and NounC targets;
(3) The sentential context helps to trigger phonological processing of the Noun, and participants’ performance will be severely interfered with on the homophone nonwords in the Noun condition but not in the NounT and NounC conditions.

## 2. Experiment 1

### 2.1. Methods

#### 2.1.1. Participants

Forty-three HanHs (36 females; *M* = 19.81 years; age range: 19–21 years) and 54 HanLs (43 females; *M* = 19.69 years; age range: 18–21 years) were recruited on the campuses of XXX University and XXX University, respectively. With more than 46 participants at each treatment level of the design, there would be a 90% power to achieve an effect size of 0.20 at the two-sided significance level of 0.05. All the participants had no hearing impairment and had normal or corrected-to-normal vision. Both groups were required to respond to a questionnaire on their language background and to attend a Chinese reading test (CRT). As indicated in Table 1, HanLs had a significantly shorter period of Chinese learning than HanHs and had a significantly lower frequency of using Chinese in activities such as dreaming, thinking, and murmuring. Moreover, their CRT scores (*M* = 16.31, *SD* = 1.77) were significantly lower than those of HanHs (*M* = 17.50, *SD* = 0.83) (*t*(95) = 5.909, *p* < 0.001, 95% *CI* = 0.79–1.59). They were less experienced than HanHs in Chinese.

In order to evaluate the potential influence of a bilingual environment on the development of HanLs’ Chinese proficiency, we conducted a post-hoc survey. Coming from communities that were physically separated from the ethnic groups in Xinjiang, the Chinese native speakers used Chinese as their sole language in daily activities before they enrolled as college students. Thirty-eight of them had no experience in Uyghur at all and the other 16 had experience in studying Uyghur in classroom activities for up to one year. They had a relatively low level of ability in listening, speaking, reading, and writing in the target language. Outside their Uyghur-learning classroom, they used Chinese as the dominant medium to communicate with others and in accessing media, for example, listening to radio, watching television, reading, surfing the Internet, or receiving/sending messages in WeChat, QQ (an electronic mode of communication similar to WeChat), and email. In short, HanLs might be largely similar to HanHs in that their Chinese language development was not heavily affected by the bilingual environment. Participants gave written informed consent in accordance with the Declaration of Helsinki. The study was approved by the Ethics Committee of XXX University.

#### 2.1.2. Materials

Fifty sentences were created, each containing an NCN, the noun of which had a character-transposed word available. A group of 22 students from the same pool as the HanHs group rated the readability of each sentence on a seven-point scale (1 = very difficult to understand; 7 = very easy to understand). The results yielded 46 sentences, each of which had an averaged readability score equal to or greater than 5.0. The noun of the NCN in each sentence was then removed to leave 46 incomplete sentences. For example, ‘妈妈有点腰疼, 叫我帮她在腰上贴了一贴 ( )’ (/ma1ma0 you3dian3 yao1teng2, jiao4 wo3 bang1ta1 zai4 yao1shang0 tie1le0 yi1tie1/, *Mum had a little backache and asked me to put a piece of ( ) on her waist*). Another 22 students were required to fill in the blanks with proper words. As a result, 24 sentences (33.42 Chinese characters in length) were obtained, each of which had a cloze probability of 80% or higher.

Following the creation of these 24 sentences, Nouns (e.g., ‘膏药’, /gao1yao4/, *plaster*) were replaced with NounTs (e.g., ‘药膏’, /yao4gao1/, *ointment*) and NounCs (e.g., ‘公鸡’, /gong1ji1/, *cock*) to create 48 more sentences. Nouns, NounTs, or NounCs in the 72 sentences were further replaced with their corresponding homophone nonwords to obtain another 72 sentences, giving a total of 144 critical sentences. Table 2 displays a set of stimuli at the six treatment levels of trgCondition and trgType.

As indicated in Table 3, there were no significant differences in frequency [18] between Nouns and NounTs (*t* = 1.280, *p* = 0.206), between Nouns and NounCs (*t* = 0.543, *p* = 0.590), or between NounTs and NounCs (*t* = 0.917, *p* = 0.364). There were no significant differences in the frequency between the Chinese characters in Nouns and the corresponding homophone nonwords (*t* = 1.413, *p* = 0.161), between those in NounTs and the corresponding homophone nonwords (*t* = 1.747, *p* = 0.108), or between those of NounCs and the corresponding homophone nonwords (*t* = −0.078, *p* = 0.938). There were no significant differences in the character frequency of the homophone nonwords between the Noun and NounT conditions (*t* = 0.332, *p* = 0.741), between the Noun and NounC conditions (*t* = 0.331, *p* = 0.742), or between the NounT and NounC conditions (*t* = 0.286, *p* = 0.977). There were no significant differences in the number of strokes between the Chinese characters in Nouns and the corresponding homophone nonwords (*t* = −1.550, *p* = 0.125), between those in NounTs and the corresponding homophone nonwords (*t* = −1.519, *p* = 0.132), or between those of NounCs and the corresponding homophone nonwords (*t* = 0.071, *p* = 0.943). There were no significant differences in the number of strokes of the homophone nonwords between the Noun and NounT conditions (*t* = 0.180, *p* = 0.858), between the Noun and NounC conditions (*t* = 0.321, *p* = 0.749), or between the NounT and NounC conditions (*t* = −0.146, *p* = 0.884).

The 144 critical sentences were divided into six equal groups, each of which contained the same number of sentences at each treatment level of trgCondition by trgType. Thirty-six filler sentences (e.g., ‘小明的爷爷是个木匠小能手,自己做了一扇纱窗,简单实用又美观’, /Xiao3ming2 de0 ye2ye0 shi4 ge4 mu4jiang4 xiao3neng2shou3,zi4ji3 zuo4le0 yi1shan4 sha1chuang1, jian3dan1 shi2yong4 you4 mei3guan1/, *Xiaoming’s grandfather is a highly skilled carpenter. He made a screen window, which looks simple, practical and beautiful*) were determined, which were similar to the critical sentences in length, readability, and syntactic structure (i.e., as in the critical sentences, each filler sentence contained an NCN). For 18 of the filler sentences, the word targets were 2C-words other than the nouns of the NCNs after the middles of the sentences. For the other 18 filler sentences, the targets were 2C-nonwords. Each of the six groups of critical sentences was randomly mixed with the 36 filler sentences to create a material block.

#### 2.1.3. Procedure

Six programs were designed using E-Prime 3.0; programs 1 to 6 were for the material blocks 1 to 6, respectively. In Experiment 1, HanHs were randomly divided into six largely equal groups assigned to a specific program. The participant was seated in front of the computer screen with their eyes 60 cm horizontally away from the center. Using a pair of earphones, the participant first heard a beep that lasted 700 ms. At the end of the auditory prompt, a red fixation cross (‘+’) was shown in the center of the screen for 500 ms. Then, the red cross was replaced by ‘…’ in a black color and, at the same time, the participant listened to the beginning part of a sentence, read in a male voice at a normal speed. When the audio recording stopped, two Chinese characters were shown in the center of the computer screen and the participant was asked to make a lexical decision by pressing one of two predefined keys on the keyboard. Once a key press was received or the target had been shown for 2000 ms, the target disappeared and the audio recording resumed. At the end of the audio, a two-choice listening comprehension question appeared pertaining to the content of the preceding sentence. The participant had to press one of two predefined keys to answer the question. Then, a blank screen was shown for 1000 ms before the next trial began. The experiment lasted half an hour. The participant received 20 yuan (2.9 USD) as a reward. Both HanLs and HanHs undertook the same procedure. The procedure in Experiment 2 was the same as in Experiment 1.

### 2.2. Results

HanHs and HanLs achieved accuracies of 0.94 (*SD* = 0.12) and 0.91 (*SD* = 0.22), respectively, in answering the sentence comprehension questions, indicating that they clearly understood the sentences. Data were removed for trials in which the RTs were shorter than 200 ms or three *SD*s above the overall average (3.9% of the collected data were discarded). Table 4 displays the results.

Binary logistic regression analyses of the raw errors (1 = incorrect response, 0 = correct response) were conducted with lme4 [20] in R [21], beginning with RawError~sbjGroup × trgCondition × trgType + (1|SubjectId) + (1|ItemID) as the formula for the generalized linear mixed model fit by maximum likelihood. Mixed-effects linear regression analyses were conducted on the logarithms of the RT scores for the correct responses. A backward selection approach was adopted to achieve the minimal adequate models. The effects results are summarized in Table 5 and Table 6. Addtional data are available upon request.

#### 2.2.1. Error Scores

The main effects were significant for sbjGroup and trgType. The two-way interactions were significant between sbjGroup and trgCondition, between trgCondition and trgType, and between sbjGroup and trgType. Simple effect analyses were conducted using the emmeans package [22]. Both HanHs (*z* = 4.337, *p* < 0.0001; *z* = 5.109, *p* < 0.0001) and HanLs (*z* = 4.296, *p* = 0.0001; *z* = 4.350, *p* < 0.0001) had a significantly lower probability of making error responses in the Noun condition (*M* = 0.003, 95%CI = 0.012; *M* = 0.017, 95%CI = 0.026) than in the NounT (*M* = 0.055, 95%CI = 0.091; *M* = 0.094, 95%CI = 0.103) and NounC conditions (*M* = 0.085, 95%CI = 0.126; *M* = 0.097, 95%CI = 0.103). Probability scores (ranging from 0 to 1) were obtained through reverse transformations of the logit scores. HanHs had a significantly lower probability of making error responses than HanLs in the Noun condition (*z* = 2.311, *p* = 0.0209).

HanHs and HanLs had a significantly higher probability of making error responses to the word targets (*M* = 0.488, 95%CI = 0.205; *M* = 0.564, 95%CI = 0.201) than to the nonword targets (*M* = 0.007, 95%CI = 0.016; *M* = 0.008, 95%CI = 0.018) in the NounT (*z* = 9.203, *p* < 0.0001) and NounC conditions (*z* = 9.677, *p* < 0.0001). The probability of making error responses was marginally significantly higher to the word (*M* = 0.015, 95%CI = 0.025) than to the nonword targets (*M* = 0.005, 95%CI = 0.013) in the Noun condition (*z* = 1.896, *p* = 0.0579). They had a significantly lower probability of making error responses to the word targets in the Noun conditions than in the NounT (*z* = 9.782, *p* < 0.0001) and NounC conditions (*z* = 10.186, *p* < 0.0001).

Both HanHs (*z* = 7.486, *p* < 0.0001) and HanLs (*z* = 8.183, *p* < 0.0001) had a significantly higher probability of making error responses to the word (*M* = 0.229, 95%CI = 0.178; *M* = 0.194, 95%CI = 0.133) than to the nonword targets (*M* = 0.002, 95%CI = 0.008; *M* = 0.014, 95%CI = 0.019). HanHs had a significantly lower probability of making error responses than HanLs to the nonword targets (*z* = 2.556, *p* = 0.0106).

#### 2.2.2. RT Scores

The main effect of sbjGroup was significant: HanHs (*M* = 854 ms, 95%CI = 109 ms) had significantly shorter RTs than HanLs (*M* = 1195 ms, 95%CI = 232 ms) (β = −0.337, *SE* = 0.043, *t* = −7.920, *p* < 0.0001). The two-way interaction was significant between trgCondition and trgType. The two groups of native speakers’ RTs to the word targets were significantly shorter in the Noun condition (*M* = 945 ms, 95%CI = 99 ms) than in the NounC condition (*M* = 1157 ms, 95%CI = 143 ms) (*t* = 6.699, *p* < 0.0001), significantly shorter in the NounT condition (*M* = 1013 ms, 95%CI = 124 ms) than in the NounC condition (*t* = 3.874, *p* < 0.0001), and marginally significantly shorter in the Noun condition than in the NounT condition (*t* = 2.346, *p* = 0.0508). Their RTs to the word targets were significantly shorter than their RTs to the nonword targets in the Noun condition (*M* = 1035 ms, 95%CI = 108) (*t* = 2.656, *p* = 0.0003), not significantly different from their RTs to the nonword targets in the NounT condition (*M* = 1056 ms, 95%CI = 110) (*t* = 1.388, *p* = 0.1659), and significantly longer than their RTs to the nonword targets in the NounC condition (*M* = 1021 ms, 95%CI = 106) (*t* = 4.132, *p* < 0.0001).

### 2.3. Discussion

HanLs had significantly longer RTs than HanHs, confirming the questionnaire data showing that they were less experienced than HanHs in Chinese and the CRT result showing that they had a significantly lower level of reading proficiency than HanHs. HanLs also had a significantly higher probability of making error responses than HanHs for the Nouns. They appeared to be less efficient than HanHs in expecting the next words in the context of audio sentence comprehension, which is probably due to their low level of Chinese proficiency.

The two groups of native speakers consistently had significantly shorter RTs for the Noun targets than for the NounC targets, and had a significantly lower probability of making error responses in the Noun condition than in the NounC condition. If the target was Noun, it matched the participants’ expectation according to the context, and a ‘yes’ response was easy to determine. If it was NounC, however, the participants might have experienced a kind of conflict. Their ‘yes’ response might have been interfered with by the semantic inconsistency between the target and the sentential context. Therefore, they performed significantly worse for the NounC targets than for the Noun targets. This finding clearly confirmed Prediction One, hereafter referred to as the sentential context effect (SCE).

The RTs of both the HanH and HanL groups were significantly shorter for the NounT targets than for the NounC targets. In agreement with Prediction Two, the native speakers had a tendency to process the 2C-word targets according to their constituent characters. They also had a similar probability of making error responses for the NounC and the NounT targets, and their RTs were marginally significantly shorter for the Noun targets than for the NounT targets. In other words, they may also have tended to take the word targets as whole entities. These two results, in combination, are compatible with the conclusion that 2C-words in isolation are processed both as whole entities and according to their constituent characters.

The finding that the HanL group had a significantly higher probability of making error responses than the HanH group in denying the nonword targets as words suggests that proficiency affects lexical skills. Interestingly, the two groups of native speakers consistently had a significantly higher probability of making error responses to the word than to the nonword targets, especially in the NounT and NounC conditions. They had significantly longer RTs to the word targets than to the nonword targets in the NounC condition. In addition to a demonstration of the SCE, these results suggest that they were quite certain of making ‘no’ responses to the nonword targets.

The native speakers’ significantly shorter RTs to the word than to the nonword targets in the Noun condition seemed to confirm Prediction Three. The preceding context may have triggered phonological representation activation for the Nouns, which matched the phonological processing of the homophone nonwords in the Noun condition. As a result, their ‘no’ responses were severely delayed.

## 3. Experiment 2

### 3.1. Method

#### 3.1.1. Participants

Fifty Korean college students (30 females; *M* = 20.12 years, age range: 18–22 years) and 37 Uighur college students (27 females; *M* = 20.3 years, age range: 18–22 years) were recruited on the campuses of XXX University and YYY University, respectively, in the same way as in Experiment 1. With at least 37 participants at each treatment level of the design, there would be a 90% power to achieve an effect size of 0.23 at the two-sided significance level of 0.05. As indicated in Table 7, Uyghurs were less experienced than Koreans in Chinese. Moreover, their CRT scores (*M* = 12.13, *SD* = 3.64) were significantly lower than those of Koreans (*M* = 16.60, *SD* = 1.65) (*t*(85) = 10.134, *p* < 0.001, 95% CI = 3.60–5.35).

#### 3.1.2. Materials and Procedure

The materials and procedure were the same as in Experiment 1.

### 3.2. Results

The data were screened and analyzed in the same way as in Experiment 1, with 4.0% of the collected data discarded. Koreans and Uyghurs achieved accuracies of 0.91 (*SD* = 0.35) and 0.85 (*SD* = 0.49), respectively, in answering the sentence comprehension questions. The descriptive results are shown in Table 8 and the effects results are summarized in Table 9 and Table 10.

#### 3.2.1. Error Scores

The main effect was significant for sbjGroup. The two-way interaction was significant between sbjGroup and trgCondition. The three-way interaction was significant between sbjGroup, trgCondition, and trgType. Koreans’ probability of making error responses was significantly lower for the Noun targets than the NounT (*t* = 6.918, *p* < 0.0001) and NounC targets (*t* = 7.611, *p* < 0.0001); Uyghurs’ probability was significantly lower for the Noun targets than for the NounC targets (*t* = 3.896, *p* = 0.0003), but was marginally significantly lower for the Noun targets than for the NounT targets (*t* = 2.285, *p* = 0.0580). Koreans’ probability of making error responses was significantly lower than that of Uyghurs for the nonword targets in the Noun (*t* = 5.569, *p* < 0.0001), NounT (*t* = 5.923, *p* < 0.0001), and NounC conditions (*t* = 5.426, *p* < 0.0001), but was significantly higher than that of Uyghurs for the word targets in the NounT (*t* = 5.616, *p* < 0.0001) and NounC conditions (*t* = 4.518, *p* < 0.0001). Koreans had a significantly lower probability for the nonword than for the word targets in the NounT (*t* = 6.767, *p* < 0.0001) and NounC conditions (*t* = 7.803, *p* < 0.0001), but Uyghurs had a significantly higher probability of making error responses for the nonword than for the word targets in the Noun (*t* = 3.645, *p* = 0.0003) and NounC conditions (*t* = 2.135, *p* = 0.0328).

#### 3.2.2. RT Scores

The main effects were significant for sbjGroup and trgType. The two-way interactions were significant between sbjGroup and trgType, and between trgCondition and trgType. Koreans (*M* = 968 ms, 95%CI = 104 ms; *M* = 1020 ms, 95%CI = 104 ms) had significantly shorter RTs than Uyghurs (*M* = 1278 ms, 95% CI= 152 ms; *M* = 1706 ms, 95%CI = 208 ms) for the word (*t* = 7.899, *p* < 0.0001) and nonword targets (*t* = 14.809, *p* < 0.0001). Both Koreans (*t* = 2.109, *p* = 0.0359) and Uyghurs’ RTs (*t* = 10.926, *p* < 0.0001) were significantly shorter for the word than for the nonword targets, but Koreans’ RT differences were significantly smaller than those of Uyghurs (β = −0.237, *SE* = 0.030, *t* = 7.898, *p* < 0.0001).

The two groups of non-native speakers had significantly longer RTs for the nonword (*M* = 1188 ms, 95%CI = 142 ms; *M* = 1191 ms, 95%CI = 144 ms; *M* = 1182 ms, 95%CI = 141 ms) than for the word targets (*M* = 993 ms, 95%CI = 116 ms; *M* = 1008 ms, 95%CI = 130 ms; *M* = 1073 ms, 95%CI = 143 ms) in the Noun (*t* = 6.473, *p* < 0.0001), NounT (*t* = 5.489, *p* < 0.0001), and NounC conditions (*t* = 3.224, *p* = 0.0016). They had significantly longer RTs in the NounC than in the Noun condition for the word targets (*t* = 2.497, *p* = 0.0363).

### 3.3. Discussion

In comparison with Uyghurs, Koreans had significantly shorter RTs to the word targets and performed significantly better on the nonword targets. These findings are consistent with the fact that they were more experienced in Chinese and achieved significantly higher CRT scores than their Uyghur counterparts. Koreans’ error responses to the Noun targets were not significantly different from those of Uyghurs, but they had a significantly higher probability than Uyghurs of making error responses to the NounT and NounC targets. In other words, Koreans and Uyghurs showed similar levels of expectation for the upcoming Noun targets in sentential contexts. Yet, while Koreans were similar to their Uyghur counterparts in taking the NounT targets as whole entities, they seemed to encounter a greater conflict than Uyghurs between sentence comprehension and ‘yes’ responses to the NounT and NounC targets.

Similar to the native speaker participants in Experiment 1, Koreans had a significantly lower probability of making error responses to the Noun target than to the NounT and NounC targets. Their error responses were not significantly different between the NounT and the NounC targets. Uyghurs were similar to Koreans in having a significantly lower probability of making error responses to the Noun targets than to the NounC targets and in having no significant differences between their response errors to the NounT and NounC targets. However, their probability of making error responses was only marginally significantly lower to the Noun than to the NounT targets. In short, they were more likely than Koreans to confuse the NounT targets with the Noun targets.

The two groups of non-native speakers had significantly shorter RTs to the Noun targets than to the NounC targets, but their RTs were not significantly different between the Noun and the NounT targets or between the NounT and the NounC targets. This finding shows that both groups tended to confuse the NounT targets with the Noun and NounC targets in RTs. Uyghurs were largely similar to Koreans regarding the influence of trgType on RT scores. Both groups of non-native speakers consistently showed significantly shorter RTs for the word targets than for the nonword targets and may have found it difficult to make a ‘no’ response to the nonword targets. This might indicate their relatively weak ability to determine whether or not a two-character cluster was a word. Since Koreans’ RT differences between word and nonword targets were significantly smaller than those of Uyghurs, they may have a superior ability to make ‘no’ responses to the nonword targets.

Uyghurs had a significantly higher probability of making error responses in their ‘no’ responses to the nonword targets than in their ‘yes’ responses to the word targets in the Noun and NounT conditions. However, Koreans had a significantly higher probability of making error responses to the word targets than to the nonword targets in the NounT and NounC conditions. It is possible that Uyghurs had a small vocabulary and found it extremely difficult to discriminate a nonword from a word, but Koreans’ responses were heavily mediated by the contexts.

## 4. Experiments 1 and 2

To compare the influence of Chinese proficiency between Experiments 1 and 2, the data were re-analyzed, with Experiment (1 or 2), sbjGroup, trgCondition, and trgType as the fixed factors. The non-native speakers of Chinese had significantly longer RTs (197 ms, 128 ms, 69 ms; 346 ms, 327 ms, 346 ms) than the native speakers in the Noun (β = 0.180, *SE* = 0.026, *t* = 6.847, *p* < 0.0001; β = 0.301, *SE* = 0.027, *t* = 11.074, *p* < 0.0001), NounT (β = 0.123, *SE* = 0.033, *t* = 3.697, *p* = 0.0002; β = 0.282, *SE* = 0.027, *t* = 10.264, *p* < 0.0001), and NounC (β = 0.060, *SE* = 0.034, *t* = 1.762, *p* = 0.078; β = 0.304, *SE* = 0.027, *t* = 11.212, *p* < 0.0001) conditions for the word and nonword targets. They also had a significantly higher probability of making error responses than their native counterparts in the Noun (β = 2.705, *SE* = 0.508, *z* = 5.327, *p* < 0.0001), NounT (β = 2.759, *SE* = 0.483, *z* = 5.710, *p* < 0.0001), and NounC (β = 2.380, *SE* = 0.493, *z* = 4.828, *p* < 0.0001) conditions for the nonword targets and in the Noun condition (β = 1.031, *SE* = 0.414, *z* = 2.488, *p* = 0.0128) for the word targets. These results directly confirmed the expectation of non-native speakers’ poorer performance in lexical processing in the context of sentence comprehension. In agreement with this argument, the non-native speakers had a significantly lower probability of making error responses than that of the native speakers in the NounT (β = 1.067, *SE* = 0.196, *z* = 5.437, *p* < 0.0001) and NounC (β = 0.545, *SE* = 0.186, *z* = 2.924, *p* = 0.0035) conditions for the word targets. In other words, the non-native speakers had a smaller conflict between their ‘yes’ responses and context comprehension than the native speakers.

## 5. General Discussion

Two experiments were conducted to examine native and non-native speakers’ recognition of 2C-words in the context of audio sentence comprehension. The recording of a sentence was played, in which a collocation composing of a number word, a sortal classifier, and a noun (NCN) was embedded. When the participants were about to hear the noun of the NCN (Noun), the playing stopped, and a target was visually presented, which was either the Noun, the character-transposed word of the Noun (NounT), or the control word (NounC), or a homophone nonword for Noun, NounT, or NounC. In Experiment 1, two groups of Chinese native speakers (HanHs and HanLs) made lexical decisions on the targets before they resumed listening. In Experiment 2, two groups of bilinguals (Koreans and Uyghurs) who learned Chinese as L2 completed the task. As expected, there were differences between Experiments 1 and 2 in the influences of Chinese proficiency on performance.

Although HanLs made significantly more error responses to the nonword targets and had significantly longer RTs than HanHs, the two groups were largely similar in confirming the three predictions. However, HanLs were less able than HanHs to predict the up-coming Noun in the experimental task, which is probably due to their lower level of proficiency in Chinese. Uyghurs performed significantly worse on the nonword targets and had significantly longer RTs for the word targets than Koreans. Both Uyghurs and Koreans were able to predict the upcoming Noun, but the former were less skillful in doing so. The two groups of non-native speakers appeared to find it difficult to discriminate between a nonword and a word, which was particularly so for the Uyghurs.

Lexical processing in Chinese sentence reading can be concurrently constrained by the prediction of the preceding context and by information regarding the likely identity of the upcoming characters [23]. As reading can be simply viewed as a combination of language comprehension and lexical decoding [24], our findings warrant further discussion.

The result of the language comprehension of the pre-texts means that the participants should have strong expectations of Nouns and may have a clear tendency to take the 2C targets as whole entities. This is why the SCE was observed in their responses across the two experiments. These results also confirm the finding of Bai et al. [11]: that words are the basic units in sentence comprehension. As a result of their lower Chinese proficiency, however, a lower level of ability in language comprehension was observed among HanLs and Uyghurs than among HanHs and Koreans, respectively.

In decoding the visually presented targets, the native speaker participants may have experienced the same processing in lexicality as in isolation [25]. They might have taken the targets both as whole entities and according to the constituent characters in the decoding procedure. As a result, their RTs were significantly longer for the NounC targets than for the NounT targets and were marginally significantly longer for the NounT targets than for the Noun targets.

According to Chinese education policy, minimum literacy requirements include the mastery of a few thousand most frequently used Chinese characters. After two or three years of literacy education, a native speaker student will be conscious of the fact that a 2C-word is composed of two characters [26]. Native speakers who are skilled readers understand a new word by combining their understandings of the constituent characters; their inevitable processing of the constituent characters in 2C-word recognition has been repeatedly confirmed in empirical studies [3,4,5]. The non-native speaker participants in Experiment 2 (for whom Chinese was L2) did not appear to process the word targets according to their constituent characters as readily as the native speaker participants in Experiment 1. For example, Koreans may not have been any less capable of sentence reading than HanLs, since they achieved similar CRT scores. However, by taking words as the basic units in their mother tongue, they may have strategically considered 2C-words as orthographic wholes in Chinese learning. As a result, their performance did not clearly indicate that they decoded the visual targets according to their constituent characters. Instead, they appeared to find it difficult to decide that a 2C-nonword was unacceptable. However, Uyghurs were more likely than their Korean counterparts to meet processing difficulties when the targets were nonwords, which was probably due to their more limited vocabulary. Their relatively weak lexical skills may also be the reason why Prediction Three was not confirmed in Experiment 2.

The implications of the present study are two-fold. The language comprehension component of reading ability is more likely than the lexical decoding component to be affected by language proficiency for adult native speakers of Chinese. However, non-native speakers’ performance in these two components of reading ability are both subject to the influence of Chinese language proficiency. While less proficient native speakers of Chinese most need to improve their language comprehension ability, less proficient non-native speakers are likely to need improvements in both lexical decoding and their language comprehension ability in written Chinese.

The present study is of significance in demonstrating the diversity of linguistic populations within one country and in revealing the contribution of a multilingual environment on the competence of native speakers. Both Koreans and Uyghurs were non-native speakers of Chinese. Due the differences in their L1 background and in L2 practices, however, the former seemed to have developed a mechanism of 2C-word processing that was obviously different from the latter. Probably largely for the sake of social–linguistic factors, HanLs appeared to have room for improvement in language comprehension in comparison with HanHs.

The present study has some limitations. According to the post-hoc survey results, the bilingual background of HanLs in Experiment 1 may have had very little effect on their Chinese proficiency. However, the results would have been more convincing if they had been from the same communities as HanHs. Similarly, in Experiment 2, if the two groups of non-native speakers had been from the same ethnic group, the results would have been more significant. Moreover, more findings would be achieved with other non-native speakers of Chinese (such as Mongolic speaking populations) as the subjects in similar studies.

## 6. Conclusions

Both native and non-native speakers are able to take visually presented 2C-word targets as semantic whole entities in the context of audio sentence comprehension, which is mediated by their Chinese proficiency. Native speakers readily process visually presented 2C-words both as wholes and according to their constituent characters, but non-native speakers are not likely to process the 2C-words according to their constituent characters. Less proficient native speakers of Chinese need to improve their language comprehension, but less proficient non-native speakers need to improve their ability in both lexical decoding and language comprehension in written Chinese.

## Figures and Tables

**Table 1:** Participants’ background information in Experiment 1.

	HanH		HanL	
	*M*	*SD*	*M*	*SD*
Activity beginning age in Chinese (year)				
Speaking	1.83	2.82	1.79	1.05
Reading	3.73	2.88	3.58	1.51
Writing	4.46	2.64	4.45	1.64
Chinese learning period (years)				
	18.28	1.63	17.14 *	2.76
Chinese self-rating score (7-point Likert scale)				
Listening	6.77	0.67	6.75	0.81
Speaking	6.77	0.73	6.69	0.89
Reading	6.72	0.88	6.68	0.84
Writing	6.79	0.69	6.59	0.95
Frequency in activities in Chinese (7-point Likert scale)				
Arithmetic	6.77	1.04	6.52	1.01
Remembering numbers	6.75	1.04	6.50	1.13
Dreaming	6.71	1.01	6.16 *	1.52
Thinking	6.75	0.93	6.38 *	1.24
Murmuring	6.75	0.84	6.26 *	1.39
Being irrational	6.67	1.08	6.31	1.28

*, *p* < 0.05.

**Table 2:** A set of stimuli at each treatment level of trgCondition by trgType.

		Pre-Text	Target	Post-Text
Noun	Word	妈妈有点腰疼, 叫我帮她在腰上贴了一贴 (/ma1ma0 you3dian3 yao1teng2, jiao4 wo3 bang1ta1 zai4 yao1shang0 tie1le0 yi1tie1/, *Mom had a little backache and asked me to put a piece of ( ) on her waist*)	膏药 /gao1yao4/ (*plaster*)	但是它的效果并不是很好。 (/dan4shi4 ta1de0 xiao4guo3 bing4 bu2shi4 hen3hao3/, *but it didn’t work very well.*)
	Nonword		糕要 /gao1yao4/	
NounT	Word		药膏 /yao4gao1/ (*ointment*)	
	Nonword		钥高 /yao4gao1/	
NounC	Word		公鸡 /gong1ji1/ (*cock*)	
	Nonword		攻积 /gong1ji1/	

**Table 3:** Attributes of the target at each treatment level of trgCondition by trgType.

		Word Frequency		Character Frequency		Number of Strokes	
		*M*	*SD*	*M*	*SD*	*M*	*SD*
Noun	Word	4.16	6.83	54.76	30.65	7.58	3.52
	Nonword	-	-	45.21	35.43	8.73	3.72
NounT	Word	2.15	3.47	54.76	30.65	7.58	3.52
	Nonword	-	-	42.78	36.34	8.60	3.05
NounC	Word	3.23	4.61	44.19	33.66	8.56	3.16
	Nonword	-	-	44.71	31.32	8.52	2.52

**Table 4:** Descriptive results of Experiment 1.

			Error Rate		RT	
			*M*	*SD*	*M*	*SD*
HanH	Noun	Word	0.02	0.15	840	324
		Nonword	0.00	0.00	879	281
	NounT	Word	0.55	0.50	891	308
		Nonword	0.01	0.08	905	294
	NounC	Word	0.62	0.49	977	311
		Nonword	0.01	0.11	871	269
HanL	Noun	Word	0.03	0.17	1178	490
		Nonword	0.03	0.18	1290	498
	NounT	Word	0.51	0.50	1239	496
		Nonword	0.03	0.16	1294	485
	NounC	Word	0.5	0.50	1405	545
		Nonword	0.02	0.15	1262	461

**Table 5:** Model coefficients (in logits) for the likelihood of producing an error in Experiment 1.

	Est.	*SE*	*z*	*p*			Variance	*SD*
(Intercept)	−6.779	0.950	−7.133	0.0000	itemID	(Intercept)	0.973	0.986
sbjGroupLow	2.614	0.885	2.953	0.0032	subjectID	(Intercept)	0.588	0.767
trgConditionNounC	1.351	0.883	1.530	0.1260				
trgConditionNounT	0.953	0.890	1.071	0.2841				
trgTypeWord	2.248	0.859	2.616	0.0089				
sbjGroupLow:trgConditionNounC	−1.485	0.680	−2.182	0.0291				
sbjGroupLow:trgConditionNounT	−1.054	0.683	−1.544	0.1226				
sbjGroupLow:trgTypeWord	−1.981	0.683	−2.902	0.0037				
trgConditionNounC:trgTypeWord	3.914	0.792	4.944	0.0000				
trgConditionNounT:trgTypeWord	3.764	0.788	4.778	0.0000				

**Table 6:** Model coefficients (in log RTs) for lexical decision latencies in Experiment 1.

	Est.	*SE*	*t*	*p*			Variance	*SD*
(Intercept)	6.752	0.036	187.691	0.0000	itemID	(Intercept)	0.004	0.067
sbjGroupLow	0.337	0.043	7.920	0.0000	subjectID	(Intercept)	0.040	0.200
trgConditionNounC	−0.013	0.025	−0.541	0.5891	Residual		0.078	0.280
trgConditionNounT	0.020	0.025	0.810	0.4187				
trgTypeWord	−0.091	0.025	−3.670	0.0003				
trgConditionNounC:trgTypeWord	0.216	0.039	5.537	0.0000				
trgConditionNounT:trgTypeWord	0.050	0.039	1.288	0.1985				

**Table 7:** Participants’ background information in Experiment 2.

	Korean		Uyghur	
	*M*	*SD*	*M*	*SD*
Activity beginning age in Chinese (year)	4.37	2.31	8.50 *	2.54
Speaking	5.58	1.81	8.66 *	2.27
Reading	6.15	2.01	8.70 *	2.27
Writing	15.94	2.89	12.30 *	2.22
Chinese learning period (years)				
	4.37	2.31	8.50 *	2.54
Chinese self-rating score (7-point Likert scale)				
Listening	5.74	1.18	5.58	1.07
Speaking	5.47	1.24	5.11 *	1.01
Reading	5.48	1.12	5.51	1.07
Writing	5.81	1.03	5.55	1.12
Frequency in activities in Chinese (7-point Likert scale)				
Arithmetic	4.39	1.84	4.85	1.64
Remembering numbers	4.96	1.86	5.55 *	1.36
Dreaming	3.50	1.92	3.05	1.73
Thinking	4.30	1.68	4.42	1.46
Murmuring	4.12	1.91	4.26	1.59
Being irrational	4.40	1.88	4.40	1.53

*, *p* < 0.05.

**Table 8:** Descriptive results of Experiment 2.

			Error Rate		RT	
			*M*	*SD*	*M*	*SD*
Korean	Noun	Word	0.05	0.21	968	332
		Nonword	0.06	0.24	1059	326
	NounT	Word	0.50	0.50	1023	332
		Nonword	0.07	0.25	1065	301
	NounC	Word	0.53	0.50	1098	331
		Nonword	0.04	0.19	1055	313
Uyghur	Noun	Word	0.07	0.26	1338	554
		Nonword	0.28	0.45	1780	597
	NounT	Word	0.19	0.39	1324	649
		Nonword	0.32	0.47	1800	593
	NounC	Word	0.30	0.46	1473	641
		Nonword	0.26	0.44	1793	578

**Table 9:** Model coefficients (in logits) for the likelihood of producing an error in Experiment 2.

	Est.	*SE*	*Z*	*p*			Variance	*SD*
(Intercept)	−3.258	0.384	−8.489	0.0000	itemID	(Intercept)	0.758	0.871
sbjGroupUyghur	2.155	0.387	5.569	0.0000	subjectID	(Intercept)	0.074	0.271
trgConditionNounC	−0.343	0.561	−0.612	0.5405				
trgConditionNounT	0.269	0.509	0.529	0.5968				
trgTypeWord	−0.009	0.535	−0.017	0.9866				
sbjGroupUyghur:trgConditionNounC	0.202	0.572	0.353	0.7241				
sbjGroupUyghur:trgConditionNounT	−0.032	0.516	−0.062	0.9503				
sbjGroupUyghur:trgTypeWord	−1.704	0.603	−2.828	0.0047				
TargetNounC:trgTypeWord	3.820	0.722	5.287	0.0000				
TargetNounT:trgTypeWord	2.886	0.682	4.231	0.0000				
sbjGroupUyghur:trgConditionNounC:trgTypeWord	−1.852	0.782	−2.369	0.0178				
sbjGroupUyghur:trgConditionNounT:trgTypeWord	−2.026	0.750	−2.702	0.0069				

**Table 10:** Model coefficients (in log RTs) for lexical decision latencies in Experiment 2.

	Est.	*SE*	*t*	*p*			Variance	*SD*
(Intercept)	6.928	0.032	218.431	0.0000	itemID	(Intercept)	0.005	0.071
sbjGroupUyghur	0.515	0.035	14.887	0.0000	subjectID	(Intercept)	0.028	0.168
trgConditionNounC	−0.006	0.032	−0.186	0.8531	Residual		0.081	0.285
trgConditionNounT	0.003	0.032	0.102	0.9188				
trgTypeWord	−0.085	0.033	−2.551	0.0119				
sbjGroupUyghur:trgTypeWord	−0.237	0.030	−7.898	0.0000				
trgConditionNounC:trgTypeWord	0.091	0.046	1.963	0.0519				

## Data Availability

The original data and analysis codes are available in the Open Science Framework (OSF) repository (https://osf.io/qxuaj/?view_only=347b906d6ec7482695dc451adf4794d9) (accessed on 27 June 2024).
